# Supplementary material for: Sex trafficking survivors’ experiences with the healthcare system during exploitation: A qualitative study
Source: PLoS One. 2023 Aug 29;18(8):e0290067. doi: 10.1371/journal.pone.0290067 (PMC10464991; doi:10.1371/journal.pone.0290067)
Supplement: S1 Appendix — (PDF) [file pone.0290067.s001.pdf]

Appendix A. Interview guide (English version)

Q1. At what point in your life, and for what duration, were you subject to sex trafficking?

Q2. Can you please describe the experience that you had with the healthcare system, while you were ‘in captivity’?

- How old were you at the time?
- What drove you to seek care?
- Where were you taken and by whom?
- What type of healthcare workers did you interact with?
- What type of diagnoses and treatments did you receive?
- Tell me about a positive/negative experience.

Q3. How did these consultations go? How were you treated?

- What do you mean “…”?
- Can you elaborate on …?

Q4. During your interaction with the healthcare system, did you inform (or try to inform) the healthcare worker that you were being trafficked and that you needed help?

- If so, what happened?

Q5. Looking back, what do you wish the healthcare workers had done, or said, during your consultation? What lessons or advice do you have for healthcare workers that may encounter people like yourself during their captivity?

Q6. Thank you again for talking with me today, is there anything else you would like to add before we finish?

Appendix B. Socio-demographic questionnaire

1. What is your age? ___________________
2. What is your gender?
    a. Male
    b. Female
    c. Gender fluid, non-binary, and/or Two Spirit
    d. Other (Please specify): _______________________
    e. Prefer not to answer
3. What is your ethnicity?
    a. White or Caucasian
    b. Black or African American
    c. Hispanic or Latino
    d. First Nation, Métis or Inuk (Inuit)
    e. Asian or Asian Canadian
    f. Other (please specify): _______________________
    g. Prefer not to answer
4. What is the highest education level that you have completed?
    a. Elementary school
    b. High School
    c. CEGEP or professional, vocational and trade school
    d. University
    e. Prefer not to answer
5. Do you identify as a visible minority?

a. Yes

b. No

c. Prefer not to answer

6. Are you a person with a disability?

Note: Person with a disability is a person who has a long-term or recurring physical, mental, sensory, psychiatric or learning impairment and: Who considers themselves to be disadvantaged in employment by reason of that impairment, or Who believes that an employer or potential employer is likely to consider them to be disadvantaged in employment by reason of that impairment; and Includes persons whose functional limitations owing to their impairment may have been accommodated in their current job or workplace.

a. Yes

b. No

c. Prefer not to answer

Appendix C. In-text citations' translation from original language to English

Participant citation in original language	Citations in English
"tu ne peux pas prendre tes propres décisions par toi-même, ta vie est gouvernée par une autre personne, puis si tu subis de la violence, la personne va te demander tout le temps t'es où, ce que tu fais, t'es avec qui"(P2)	"you can't make your own decisions, your life is governed by another person, and if you are being abused, the person will always ask you where you are, what you are doing, and who you are with" (P2)
"si t'es à terre, il n'a pas le choix de t'amener voir le médecin. Sinon t'en fais plus d'argent." (P5)	"If you're run down, he has no choice but to take you to the doctor. Otherwise, you don't make money,"(P5)
"le lien de confiance c'est important, parce que souvent ces filles-là, elles n'en n'ont pas"	"the trusting relationship is important, because often these girls don't have any" (P1)
"Le gars n'ira pas attendre six heures à l'hôpital avec toi, fait que tu vas y être toute seule. Fait que c'est en plein le bon moment pour essayer d'amener la personne à faire quelque chose"(P4)	"The [trafficker] is not going to wait six hours in the hospital with you, so you're going to be there by yourself. That makes it the right time to try to get the person to do something"(P4)
Parce que j'avais une confiance en eux parce que j'y allais déjà sur une base régulière pour faire des tests de MTS ou pour avoir comme des préservatifs (…) J'étais honnête, je leur ai dit dès que j'ai commencé [le travail du sexe]. Il n'y avait pas de jugement de leur part (P2	"Because I trusted them because I was already going there on a regular basis to get STD tests or to get like condoms (…) I was honest, I told them as soon as I started [sex work]. There was no judgment on their part" (P2).
"Tu sais tu t'auto-flagelle toi-même, fait qu'en plus le jugement des autres, on oublie ça." (P7)	"You already self-flagellate yourself, so the judgment of others on top of it, forget about it." (P7).
"ça défait un peu le portrait de ton allié puis chose quand finalement il veut te coucher-, après ça il dit qu'il veut t'aider, fuck you. Genre… t'es un parmi d'autres."	"It kind of defeats the purpose of your ally when he wants to sleep with you and then he says he wants to help you, f-you. Like... you're like another one of them." (P7)
"le fait qu'ils côtoient beaucoup ce genre de clientèle-là aussi, c'était plus propice à… t'avais pas besoin d'en parler, ils le savaient. "(P7)	"The fact that they are in contact with this type of clientele [sex workers], was more conducive to... you didn't need to talk about it, they knew it" (P7)
Les gens qui n'ont pas conscience puis qui ne sont pas ouverts à la situation puis qui ne savent pas que ça se peut, (…) c'est sûr que tu peux pas deviner des choses que t'est pas conscient que ça existe (P1)	"Those who are not aware [of sexual trafficking] and who do not know that it can happen, (...) it is certain that you cannot guess things when you are not aware that they exist" (P1)

"Quand tu dis travailleuse du sexe, dans leur tête c'est volontaire" (P2)	"When you say sex worker, in their minds it's voluntary" (P2)
"hey t'as-tu un pimp?"	"Do you have a pimp?" (P1)
"Posez-leurs la question. La seule affaire qui peut arriver c'est vous allez l'insulter. Puis au moins t'auras pas passé à côté (…) ça se peut que tu sauves quelqu'un"	"Ask them the question. The only thing that can happen is that you will insult them. At least you won't have missed something (...) you might save someone" (P5)
Je me mentais de même, parce que je pense qu'au début c'est ça que les personnes que tu travailles pour eux te font croire, mais c'est sûr qu'avec le temps (….) tu leur donne quand même la moitié de ton argent, tu donnes plus que la moitié de ton argent puis c'est sûr qu'à moment donné ils te font vivre des choses-, bien avec le temps, je pense c'est devenu de l'exploitation mais même moi au début je pensais que c'était du travail du sexe (P2).	"I was lying to myself as well, because I think that in the beginning that's what the people you're working for make you believe, but it's sure that with time (....) you give them half of your money, you give more than half of your money and then it's sure that at a certain moment they make you live things-, well with time, I think it became exploitation but even me in the beginning I thought it was sex work" (P2).
Originally in English	"I think having a good support program, you tailor it to each individual, try to figure out what's their home situation with their family like, you know, is there any resources that them or their family could have" (P3)
Originally in English	"it's a matter of working with that individual, that person" (P3)
Originally in English	"For me to trust someone like, I would rather run back to my pimp than have to go through so many different loopholes just to get where I need to get to be safe. I would rather just go back there because I know I have a place to stay, I know I'll have food, and I know I'll have like certain things. Yeah, I might have to be abused but I don't want to deal with uncertainty. I want to go into a situation feeling safe and confident. (P3)"

Appendix D. Protocol for managing distress in the context of a research interview

(Modified from : Draucker C B, Martsolf D S and Poole C (2009) Developing Distress Protocols for research on Sensitive Topics. *Archives of Psychiatric Nursing* 23 (5) pp 343-350)

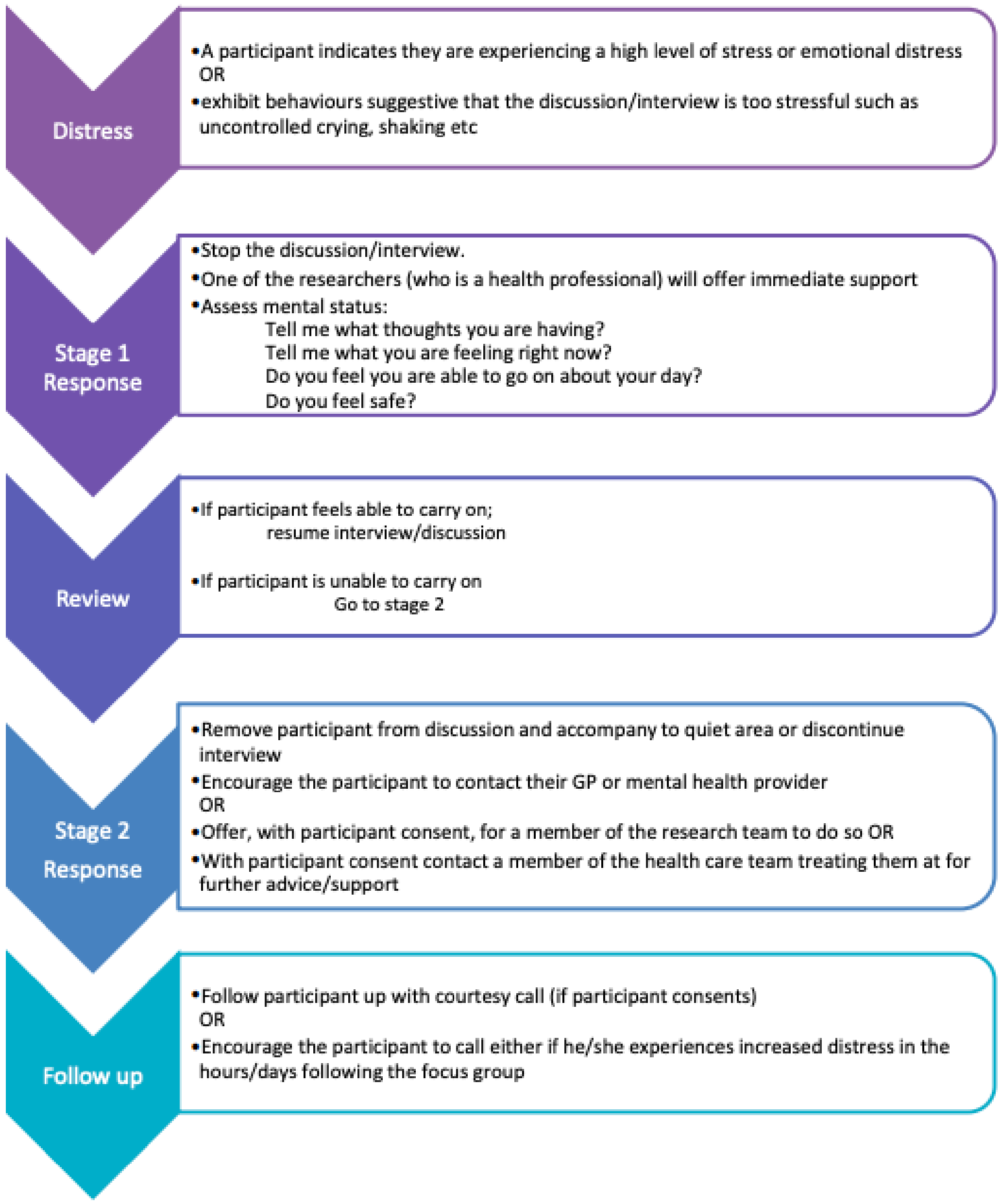

(Haigh & Witham, 2013).

Appendix E. Advice from survivors' accounts to approach and intervene with sex-trafficked individuals

Survivors' advice	Quotes
**To Approach**	
Given the opportunity, conduct a thorough physical assessment.	"You know a good inspection of the body, when you go into your patient's room you look at them from head to toe, there are also signs, you know why the patient has a burn on their arms, why they have this" (P1)
Recognize the following cues to trafficking:	"Recognize the signs that someone is being trafficked, or someone is being abused, or exploited" (P3)
• Signs of abuse	"I was strung up from the head, I was struck, I was beaten often, I had many scars" (P1)<br>"I was locked in a bathroom for 2 days, I hadn't eaten anything but soda crackers. When I got out of there, I was beaten" (P1)<br>"you usually know when someone is being trafficked because there's pretty much obvious signs, like domestic abuse" (P3)<br>"A girl who is full of bruises, who comes with a miscarriage, you know it's probably due to the blow I had a few days before" (P4)<br>"I went to the CLSC the first time to see if I had been raped, it's certain that something happened" (P6)
• Dressing	"People shouldn't be prejudiced but I looked more like a stripper than a person who works in an office " (P4)<br>"Sure when you show up in high heels and a low cut, you know I mean, it's not a judgment but the connection is easy to make I think" (P7)
• Dynamics between victim and exploiter when together	"For example, what's happened with me where I've been in the room with my abuser, and I kept looking over at him for answers of like am I giving the right answer?" (P3)<br>"they are often looking out to make sure they keep an eye on their abuser or they refuse to answer certain questions, or they are looking for answers or they are making sure that they are answering the questions right" (P3)

• Emotional state and behaviors	"They might be extremely, extremely quiet and not talk " (P3)<br>"I was always checking my cell phone, you know, I was stressed, I was scared, I didn't know what to do, they were asking me questions, I was saying banal things you know? But I was still wary." (P4)<br>"you don't have someone who walks into the office and says I have AIDS, crying uncontrollably, 19 years old and then has no reason." (P5)
• Paying with cash; no insurance card	"He threw me $200 and then told me to go to the clinic and make arrangements." (P5)<br>"I no longer had a health insurance card" (P5)
• Drug use	"C’est sûr que le fait que j’étais toujours gelée" (P7)
• Exploitative working conditions	"I suffered like psychological violence and the environment of the people you are with; you give them half of your money, you give more than half of your money and it's sure that at some point they make you do things - with time, I think it became exploitation " (P2)
Approach with a non-judgmental, respectful, and open attitude.	"Well I really think that if we don't feel judged, if we feel that the person can understand and then they listen and we see that they would be able to listen to us, maybe with the questions that they ask or the approach that they have with us, I think that maybe that would make a difference and we would feel that you know, let's say that the nurse is young and everything and starts talking to me you know, like between two girls" (P4)<br>"If you talk to her like a human, like you would talk to your sister, your friend, a normal lady, well maybe she will say my God, he respects me, so maybe she will be more willing to open up. " (P5)<br>"Do active listening, being there for them. Take the time to take the time." (P7)

Ask pertinent psychosocial questions based on cues observed. If in doubt, ask.	"Maybe if someone had asked me, do you experience this, do you experience this at home, I might have said 'no, it's not normal that I am experiencing this'".(P1)<br>"You know just talk, you don't have to ask direct questions like do you have a pimp" (P1)<br>"who do you work for, how many hours do you work, did they ask you to do things that you didn't want to do, what is your reality in terms of the money you make?" (P2)<br>"asking questions is very important, stop, try to analyze the case " (P5)<br>"I just want you to know that let's say the day the guy is going to take your money that hey maybe it's not normal... that when you're whoring all night, you're making clients and then you have $10 or $20 left in your wallet, there's something wrong. You know? I think it's more this approach of ringing bells, without judgment, that's really the key " (P7)
Conduct a routine danger/abuse assessment for sex workers.	"Because they don't ask you if you want to stop every time, or if you're in danger .... if there are people around you that can make you see that maybe you're not in the right track or whatever, you know, ask the girls if they're okay with their situation, if they're in danger. You know the first time you're not going to say yes but at some point if you trust the girl and at every appointment she asks you if you're in danger, if you've experienced violence, if you feel like you're being exploited maybe that could improve the experience" (P2).
**To Intervene**	
Offer resources to seek help and exit.	"help us feel that we have a safe place to go is just something that they could do" (P3)<br>"just provide information about existing services, even if the girl didn't disclose to you that she was a victim of trafficking" (P4)<br>"give them a condom with a 24-hour phone number where they can get a call, or tell them we're not far away, call us" (P6)

Respect survivors' choice and agency during the exiting process.	"90% of the time it probably won't work, but if 5% of the time it does, well, you've done your job. Because your job is to help people, not to leave them in the worst state" (P5)<br>"I mean you can't force them to leave, but at least offer them help, ask questions" (P5)<br>"It's to welcome them during their crisis and then even if she goes back in there, she's going to go back. chances are she's going to go back. But to just be there, next time, then to be there for her." (P7)
Understand that exiting is not a linear trajectory and takes time.	"I would say that it took about like two years to get out of the sex trafficking, but I find that if the reception was better with the health system, or them being able to pick up on signs of what was going on, like I might have been able to get out of it sooner. " (P3)
Recognize conflicting biases/beliefs and ensure continuity of care regardless	"Keep it ethical, keep it clean, you know, as much as possible and if it is something that a caretaker or healthcare worker or doctor just simply doesn't care, at least maybe transfer them to somebody who does care, or can help them." (P3)
Tailor interventions to survivors' strengths and resources.	"Good support program, you tailor it to each individual, try to figure out what their home situation with their family, like you know, is there any resources that them or their family could have" (P3)
Assess the readiness to leave trafficking/sex work	"The person also has to be ready too to get out. Like there has to be willingness in the person too" (P3)
Offer concrete resources, care, and exit pathways to address needs.	"We are scared, and we are in a position that we have to leave everything that we know or we go back. You know? We have to be completely removed from the situation or we are going to go back." (P3)<br>"For me to trust someone like I would rather run back to my pimp than have to go through so many different loopholes just to get where I need to get to be safe, I would rather just go back there because I know I have a place to stay, I know I'll have food, and I know I'll have like certain things. Yeah, I might have to be abused but I don't want to deal with uncertainty. I want to go into a situation feeling safe and confident, I don't want to, I think that's another reason why a lot of girls get out, or guys, they don't really leave because there's nothing really concrete set into what kind of help or what resources or which way they can get out safely." (P3)

Offer short-term and long-term psychological support	"I think like, you really need support with the drug addictions counselling, and the psychological impact after coming through stuff like that is very overwhelming, and since they really get to you, it's much harder to get out and start to live a normal life. Just because they destroyed every single thing, every single part inside of you that you felt good about… " (P3)<br>"all of that crazy stuff happened within a year period and like the recovery was up until, it took about two years, three years before I was able to kind of function normally." (P3)<br>" would say to you even more the prostitution itself it is damages that you realize afterwards. Until three years ago I had nightmares, all that... so that's something that at that time you don't realize all the consequences, the impact that it has on your future." (P7)
Offer interdisciplinary care and collaborate with other social services.	"There should be a good way to have even the health system aware of all these things and to be able to collaborate with law enforcement or other services that they have in order to help people. " (P3)
Ensure continuity of care	"I had a good worker but it changes, there's such a turnover, that's a big gap, you change social workers, case workers all the time" (P7)
Offer 24/7 access to resources for trafficked people	"There should be 24/7 responders, Friday night at 3am when you've just done a client and then you have no one, you have to go back to the car, you have to go back to your pimp, you know?" (P7)
Need for a network of specialist in trafficking	"I think that there is a big gap and that there should perhaps be networks of psychologists who are more informed about [trafficking] and who can receive this type of person who has lived through this and that they can help them, that they are better equipped, better able to help them because they are specialized in this." (P4)

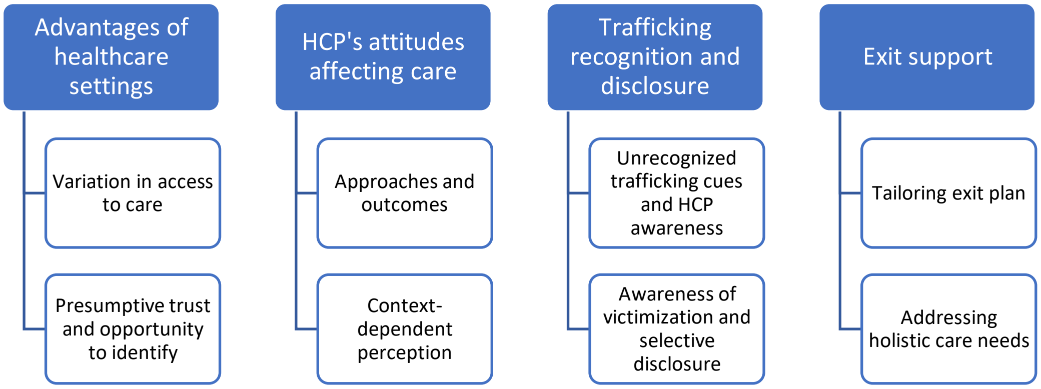
Advantages of healthcare settings
Variation in access to care
Presumptive trust and opportunity to identify
HCP's attitudes affecting care
Approaches and outcomes
Context-dependent perception
Trafficking recognition and disclosure
Unrecognized trafficking cues and HCP awareness
Awareness of victimization and selective disclosure
Exit support
Tailoring exit plan
Addressing holistic care needs
