# Supplementary material for: Sex trafficking survivors’ experiences with the healthcare system during exploitation: A qualitative study
Source: PLoS One. 2023 Aug 29;18(8):e0290067. doi: 10.1371/journal.pone.0290067 (PMC10464991; doi:10.1371/journal.pone.0290067)
Supplement: Attachment — Submitted filename: Response to reviewers.docx [file pone.0290067.s003.docx]

**Response to reviewers**

Thank you for the giving us the opportunity to submit a revised draft of our manuscript. We are grateful for the time and effort that you dedicated to give us insights and feedback. We have incorporated most of the suggestions you made. Please find point-by-point answers to comments below. Revisions in the manuscript are marked using the “track changes” feature in Word.

1) Abstract: Second sentence of paragraph three uses the term "investigative examination". I am not sure exactly what the authors mean here (forensic, comprehensive)? But I believe the term investigative denotes that healthcare providers are investigating situations surrounding human trafficking or violence, which is not part of a healthcare providers' role. I would recommend changing this term to more accurately reflect the role of healthcare providers.

**As suggested, the word investigative was replaced by comprehensive.**

2) Introduction - first paragraph: Human trafficking is an umbrella term encompassing labor trafficking, sex trafficking, organ trafficking, forced marriage, etc. The statistics from ILO include all of these different forms of trafficking, yet they are presented as only representing sex trafficking. Please provide a sentence noting the broad definition of human trafficking and stating that this paper will focus exclusively on sex trafficking.

**Thank you for this suggestion. In the first paragraph, we mention that the ILO statistics represent human trafficking, not sex trafficking:** “According to the International Labour Organization’s (ILO) most recent and rather conservative estimates, 89 million people were victims of human trafficking worldwide between 2012 and 2017, with 40.3 million actively being trafficked in 2016.”. **However, in Canada, the number of police-reported cases are mostly sex trafficking, which we define later: “**In Canada, the consensus is that human trafficking in and through the country is much more prevalent than suggested by previous estimates or the 1700 police-reported cases from 2009–2018, of which commercial sexual exploitation or sex trafficking is the largest subset.” **As the other forms of trafficking are not as present in Canada according to reports and since the paper focuses on sex trafficking, we chose not to give the broader definition of human trafficking which has been widely discussed in the literature.**

3) Page 6, line 96-98 - The abbreviation HCP is used before it is introduced and then is not used consistently throughout the paper.

**Thank you for pointing this out. This was revised.**

4) Page 6, line 102-103 - The term "escape" is used here. As the remainder of this paper focuses on survivor self-agency on determining what is best, consideration should be given to avoid the narrative of "rescuing" human trafficking victims by using terms like "escape" and instead focused on providing them the resources and support they desire.

**We think this is an excellent suggestion. The word escape was replaced with exit.**

5) Page 7, line 127 - The term "she" is used to describe a sex trafficking survivor. I would consider avoiding gendered language or provide justification for it's use.

**As suggested, we replaced “she” with “they”.**

6) Page 7, line 130 - Change "author's knowledge" to "authors' knowledge"

**Thank you for pointing out this typo. We have corrected it.**

7) Page 7, line 133 - There is a great deal of data to refute current rankings or hotspots of human trafficking because of the poor prevalence data available internationally (due to the reasons you note in the introduction). Please use caution with the term "hotspot" and consider providing justification based on data.

**We agree that the term should be removed. This was changed to “a destination for sexual tourism in North America”.**

8) Page 11, Ethical considerations - Were participants offered resources or support if the interview process brought up emotions or concerns that they wanted to discuss with someone?

**Yes, we added the following to highlight this fact: “**Care was taken to prevent and mitigate distress during the interview (see Appendix D in S1) and participants had access to mental health care through the survivors’ program. They were strongly encouraged to utilize them if they needed additional support post interview**.”**

9) Table 2 - please provide a key to define RAMQ

**This was removed entirely and replaced by “Quebec health insurance card”.**

10) Page 20, line 348 - The abbreviation LCHC is never used again. I would remove it.

**As suggested, this was removed.**

11) Page 28, line 501 - Should "exiting services" be "existing services"?

**This was meant to be “exiting services” and so it was left as is.**

12) Page 344, line 619 - Change "All participant accessed" to "All participants accessed"

**Thank you for pointing out this typo. We have corrected it.**
